# Phenotypic Responses to a Lifestyle Intervention Do Not Account for Inter-Individual Variability in Glucose Tolerance for Individuals at High Risk of Type 2 Diabetes

**DOI:** 10.3389/fphys.2019.00317

**Published:** 2019-03-26

**Authors:** Grainne O’Donoghue, Aileen Kennedy, Gregers S. Andersen, Bernadette Carr, Stephen Cleary, Eoin Durkan, Heidi Davis, Kristine Færch, Paula Fitzpatrick, Helena Kenny, Noel McCaffrey, Javier Monedero, Enda Murphy, John Noone, Tommi Suvitaival, Tanja Thybo, Michael Wheeler, Dorte Vistisen, John J. Nolan, Donal J. O’Gorman

**Affiliations:** ^1^School of Health and Human Performance, Dublin City University, Dublin, Ireland; ^2^Steno Diabetes Center Copenhagen, Gentofte, Denmark; ^3^VHI Healthcare, Dublin, Ireland; ^4^Trinity College Dublin, Dublin, Ireland; ^5^National Institute for Cellular Biotechnology, Dublin City University, Dublin, Ireland

**Keywords:** lifestyle intervention, risk of type 2 diabetes, glucose tolerance, inter-individual variability, prevention

## Abstract

**Background:** Lifestyle interventions have been shown to delay or prevent the onset of type 2 diabetes among high risk adults. A better understanding of the variability in physiological responses would support the matching of individuals with the best type of intervention in future prevention programmes, in order to optimize risk reduction. The purpose of this study was to determine if phenotypic characteristics at baseline or following a 12 weeks lifestyle intervention could explain the inter-individual variability in change in glucose tolerance in individuals with high risk for type 2 diabetes.

**Methods:** In total, 285 subjects with normal glucose tolerance (NGT, FINDRISC score > 12), impaired fasting glucose (IFG) and impaired glucose tolerance (IGT) were recruited for a 12 weeks lifestyle intervention. Glucose tolerance, insulin sensitivity, anthropometric characteristics and aerobic fitness were measured. Variability of responses was examined by grouping participants by baseline glycemic status, by cluster analysis based on the change in glucose tolerance and by Principal Component Analysis (PCA).

**Results:** In agreement with other studies, the mean response to the 12 weeks intervention was positive for the majority of parameters. Overall, 89% improved BMI, 80% waist circumference, and 81% body fat while only 64% improved fasting plasma glucose and 60% 2 h glucose. The impact of the intervention by glycaemic group did not show any phenotypic differences in response between NGT, IFG, and IGT. A hierarchical cluster analysis of change in glucose tolerance identified four sub-groups of “responders” (high and moderate) and “non-responders” (no response or deteriorated) but there were few differences in baseline clincal and physiological parameters or in response to the intervention to explain the overall variance. A further PCA analysis of 19 clinical and physiological univariables could explain less than half (48%) of total variability.

**Conclusion:** We found that phenotypic characteristics from standard clinical and physiological parameters were not sufficient to account for the inter-individual variability in glucose tolerance following a 12 weeks lifestyle intervention in inidivuals at high risk for type 2 diabetes. Further work is required to identify biomarkers that complement phenotypic traits and better predict the response to glucose tolerance.

## Introduction

Several large clinical trials have shown that the onset of type 2 diabetes can be prevented or delayed among adults at high risk by a combination of diet and exercise (Tuomilehto et al., 2001; Knowler et al., 2002; Lindström et al., 2006; Ramachandran et al., 2006; Li et al., 2008). Even so, individual responses to lifestyle interventions are variable and further investigation is required to optimize risk reduction strategies. A better understanding of the variability in physiological responses would help match individuals with the best type of intervention in personalized prevention programs (Glauber and Karnieli, 2013; Stefan et al., 2016).

One of the factors that could influence the response to a lifestyle intervention in pre-diabetes is the underlying pathophysiology (Faerch et al., 2016). Previous diabetes prevention programs included mainly subjects with Impaired Glucose Tolerance (IGT) whereas few studies have included subjects with Impaired Fasting Glucose (IFG) or Normal Glucose Tolerance (NGT) who were nonetheless at high risk for progression. Differences in the physiological responses to the same exercise or dietary interventon, or indeed the type and amount of either, could also account for variability (Bohm et al., 2016). These factors may explain why some parameters, for example body fat, may improve following a lifestyle intervention while others may be unchanged or even deteriorate (Stephens and Sparks, 2015; Sparks, 2017).

The principal aim of the DEXLIFE (Diet and Exercise for Life) project is to identify novel biomarkers that complement clinical and physiological variables to better predict improvements in glycemic status following a lifestyle intervention (Andersen et al., 2014). As a first step, a 12 weeks lifestyle intervention was designed to investigate the range of physiological responses in a group of individuals who were at risk for type 2 diabetes (O’Donoghue et al., 2015). The purpose of this study was to determine if phenotypic characteristics at baseline or following a 12 weeks lifestyle intervention could explain the inter-individual variability in glucose tolerance in high risk individuals for type 2 diabetes.

## Materials and Methods

### Setting

The study was conducted at Dublin City University (DCU), Ireland. The DEXLIFE intervention (12 weeks lifestyle program) was delivered in Dublin City Sport, an on-campus gym. This study was carried out in accordance with the recommendations of Declaration of Helsinki with written informed consent from all subjects. The protocol was approved by the Research Ethics Committee at DCU (DCUREC/2012/080) and all subjects provided written informed consent.

### Participant Eligibility

Adults aged 18–75 years, who were inactive (<150 min of physical activity per week) and displaying at least one of the following diabetes risk factors were eligible to participate; (i) impaired fasting glucose (FPG levels ≥5.6 to <7 mmol/L (ii) impaired glucose tolerance (2 h plasma glucose levels ≥7.8 to <11.1 mmol/L following an oral glucose tolerance test) and/or (iii) normal glucose tolerance with a FINDRISC (Lindstrom and Tuomilehto, 2003) score >12 (1 in 6 chance of developing type 2 diabetes in the next 10 years). Individuals were excluded if they had previously diagnosed type 2 diabetes, severe cardiovascular, respiratory or renal disease, active cancer, neuromuscular, musculoskeletal or rheumatoid disorders exacerbated by exercise, significant cognitive or mental illness, if they were receiving any medication that could affect glucose metabolism, if they had a peak aerobic capacity >50 ml^.^kg^-1.^min^-1^ or >5% change in body weight in the previous 3 months.

### Recruitment

Participants were identified in three ways. Information sessions were held locally; within the university, in local sports clubs, pharmacies and general practices within a 10 km radius of Dublin City University. An online screening tool (FINDRISC) was accessible on the DEXLIFE website. If an individual scored >12, an email was automatically generated to the DCU recruitment team and the potential participant contacted. Finally, Vhi Healthcare, Ireland’s largest health insurance company, and one of the partners in DEXLIFE, identified eligible participants from their database of policy holders. All potential participants were provided with research study information sheets and consent forms.

### Procedures

At baseline and following the 12 weeks lifestyle intervention, participants completed a number of clinical and physiological assessments.

#### Anthropometrics

Body weight was assessed on a digital platform with minimal clothing, and height was recorded on a stadiometer (SECA, Hamburg). Dual X-ray absorptiometry (Stratos, BMD Medical Systems) was used to quantify total body fat and fat-free mass while subcutaneous and visceral fat depth was measured by ultrasonography (Aquila, Pie Medical).

#### Glucose Tolerance

A standard 75 g Oral Glucose Tolerance Test (OGTT) was performed in the morning after an overnight fast. Baseline blood samples were taken for glucose, insulin and lipids followed by samples at 30, 60, 90, 120, and 180 min post-glucose ingestion. The area under the glucose curve (AUC_glucose_) and insulin (AUC_insulin_) were calculated using the trapezoidal method. Insulin secretion was estimated by the insulinogenic index (Goedecke et al., 2009) and the insulin AUC from 0 to 30 min while insulin sensitivity was estimated by the Matsuda index (Matsuda and DeFronzo, 1999).

#### Cardiorespiratory Fitness

A 12-lead ECG stress test using a modified Bruce protocol was used to assess maximal oxygen consumption (VO_2_max). Participants walked on a treadmill with either the speed or gradient increasing every 3 min until volitional fatigue or symptoms that warranted termination. Blood pressure was taken at each stage and heart rate was measured continuously. Oxygen consumption was measured using breath-by-breath analysis of expired air by indirect calorimetry (Vmax 29C, SensorMedics, Yorba Linda, CA, United States).

#### Laboratory Analyses

Serum insulin was measured with a commercially available fluoroimmunoassay (Roche Diagnostics, Mannheim, Germany). Plasma glucose was measured using a glucose oxidase method (Randox Laboratories, Crumlin, Co. Antrim, United Kingdom). Serum triglycerides, total cholesterol, HDL-cholesterol and LDL-cholesterol were measured using enzymatic methods (Randox Laboratories, Crumlin, Co. Antrim, United Kingdom).

### Lifestyle Intervention

The DEXLIFE lifestyle intervention was a 12 weeks supervised exercise training program accompanied with dietary advice.

#### Exercise Program

Participants were given access to DCU Sport. A qualified sports scientist or physiotherapist accompanied each individual to the gym for an induction session prior to commencing the intervention. The induction session included familiarization with the gym equipment and specific individual instruction relating to frequency, intensity, time and type of exercise to be performed. Participants performed 4 × 45 min exercise sessions per week at a moderate intensity, focusing on a combination of cardiovascular and resistance exercise. Exercise supervision was provided by the gym instructors based in DCU Sport. They were present during the exercise sessions, answered any questions and provided support to assist participants achieve the optimal exercise intensity. A personal online exercise diary was also made available for participants to track their individual progress and record any additional information, including other exercise.

#### Dietary Advice

A 3 day estimated food diary was used to assess dietary intake. Once completed, the participant met with a dietician to review the diary, identifying unhealthy food choices and to develop a plan to modify those choices. The concept of energy balance and restricting energy intake from fat was introduced. The energy goals were calculated by estimating the daily calories needed to maintain the participant’s starting weight and if weight loss was indicated, 500–1000 calories were subtracted per day (depending on body weight) to achieve a 0.5 kg decrease in weight per week. Common to all food plans was <10% energy intake from saturated fat intake as well as a dietary fiber intake of >15 g/1000 kcal.

#### Adherence

To optimize adherence, an electronic exercise diary was employed and regular follow-up telephone calls were used. Participants were asked to record all exercise sessions in the diary, providing details of the frequency, time, intensity and type of exercise completed. Alongside the electronic diary, participants signed in each time they attended the gym and this information was provided to the research team. Participants were weighed by a gym instructor on a weekly basis in DCU Sport and they then entered their weight into their electronic exercise diary. The research team monitored the diary entries closely and contact was made if the diary was not completed for more than 2 days in a row or if body weight was not decreasing. Adherence rates were based on the number of completed exercise sessions with 100% adherence being 48 sessions (4 sessions × 12 weeks).

### Data Analysis

The proportion of missing data ranged from <5% to 20% for the covariates. To avoid exclusion of participants with missing values which may infer biased results (Janssen et al., 2010), missing data on the covariates were imputed using the Multivariate Imputations by Chained Equations (MICE) method (van Buuren, 2007) with missing-at-random assumptions (R software). Fifty copies of the data, each with missing values suitably imputed, were independently assessed in the analyses described below. Estimates of parameters of interest were averaged across the copies according to Rubin’s rules (Marshall et al., 2009).

In order to investigate the variability in response to the lifestyle intervention, a number of analytical steps were included. The first step was to determine the impact of the intervention on the group as a whole using Paired *t*-tests. The second step examined individual variability for each of the parameters. Participants were ranked and sorted according to the change from baseline for each parameter and the individual responses were represented as waterfall plots (Gillespie, 2012).

Step 3 grouped participants by their baseline glycaemic status; NGT, i-IFG, IGT, or screen-detected type 2 diabetes (T2DM). Mean values of change with 95% CI for the covariates were calculated and the corresponding differences between groups were tested in a linear regression analysis, adjusted for age, gender, BMI and the baseline level of the covariate.

Step 4 examined the change in glucose tolerance (AUC_glucose_) by dividing participants into clusters of similar response. We used a hierarchical clustering approach based on the Euclidean distance (the absolute difference in change in outcome variables) between the observations and forming clusters using the Ward’s method (Hastie et al., 2009). The Ward’s method forms clusters where the total within-cluster variance is minimized (compact clusters), and tends to produce clusters of more equal size than others. Linear regression analysis was conducted on each of the parameters at baseline firstly to determine if it was possible to predict responsiveness to the intervention from the change in glucose tolerance and secondly to identify differences in the response to the intervention for body composition, clinical parameters and fitness between the clusters.

With the final step, we further explored the individual responses using unbiased principal component analysis (PCA) to summarize and visualize the responses to all the observed variables (Zhang and Castello, 2017). The multivariate data was grouped into principal components in an attempt to identify the parameters that account for most of the variance. Statistical analyses were performed in R version 3.3.1 (The R Foundation for Statistical Computing)^1^.

## Results

### Participant Characteristics

Of the 285 participants recruited to the lifestyle intervention, 28 (9.3%) did not participate in the follow-up examination, leaving 257 for analysis. At baseline, the mean age was 54.2 ± 10.8 years with half of the participants being female (50.2%; *n* = 129). Almost half of the participants (48.2%; *n* = 123) drank alcohol but only 8% were smokers (*n* = 20). Participants were excluded if they were receiving any medication that could affect glucose metabolism but 98 participants (38.2%) were taking other prescribed medication at the time of the intervention. The most commonly prescribed were anti-hypertensive (18.6%; *n* = 46), lipid lowering (15.4%; *n* = 39) and analgesic (8.7%; *n* = 22) medication. Of these, 22.2% were taking both anti-hypertensive and lipid lowering medication and 9.8% were taking all three.

The exercise programme adherence rate was high, with participants completing 46.2 ± 8.0 of the prescribed 48 exercise sessions (96%). Several beneficial changes in clinical and metabolic parameters were observed following the 12 weeks lifestyle intervention (Table 1). As expected, there was a significant reduction (*p* < 0.001) in a broad range of parameters including body weight (-3.9 kg: 95% CI -4.3; -3.4), waist circumference (-5.1 cm: 95% CI -6.1; -4.1), body fat (-2.0%: 95% CI -2.3; -1.7), fasting (-0.2 mmol/l/l: 95% CI -0.28; -0.13) and 2 h glucose (-0.48 mmol/l/l: 95% CI -0.70; -0.26). There were no significant differences in HDL cholesterol and fasting insulin. We also had a parallel but not randomized group (*n* = 80) that were provided with physical activity and dietary recommendations. This data is not presented as the focus of this paper is to examine variability within the intervention group and provided guidelines was itself an intervention. However, the pre and post-data from this group are presented in Supplementary Material to demonstrate that most of the physiological variables did not change, in line with findings from previous diabetes prevention studies.

**Table 1:** Baseline characteristics of the study population (original data) and estimated impact of intervention (imputed data).

	Original data		Imputed data	
		
	n	Level	Change (95% CI)	*P*
Age (year)	257	54.2 (10.9)		
Male sex (%)	257	49.8		
**Body composition**				
Weight (kg)	257	89.7 (17.9)	-3.9 (-4.3; -3.4)	<0.001
BMI (kg/m^2^)	257	31.1 (5.5)	-1.3 (-1.5; -1.2)	<0.001
Waist (cm)	224	104.4 (12.4)	-5.1 (-6.1; -4.1)	<0.001
Fat %	257	37.8 (8.6)	-2.0 (-2.3; -1.7)	<0.001
Subcutaneous fat (cm)	216	2.6 (1.97; 3.52)	-0.45 (-0.58; -0.32)	<0.001
Visceral fat (cm)	203	7.09 (5.72; 8.56)	-1.04 (-1.34; -0.74)	<0.001
**Clinical measurements**				
Fasting plasma glucose (mmol/l)	254	5.88 (0.87)	-0.20 (-0.28; -0.13)	<0.001
2 h plasma glucose (mmol/l)	249	6.83 (2.24)	-0.48 (-0.70; -0.26)	<0.001
Insulin (pmol/l)	255	76.6 (49.6; 116.9)	-8.1 (-19.6; 3.32.0)	0.164
AUC glucose (mmol min/L)	227	1158 (1002; 1344)	-65.0 (-87.4; -42.7)	<0.001
AUC insulin (⋅10^3^ pmol min/L)	237	73.0 (48.8; 106.2)	-19.4 (-23.6; -15.3)	<0.001
Matsuda index	232	2.9 (2.0; 4.6)	0.72 (0.49; 0.96)	<0.001
Insulinogenic index	257	113.5 (70.4; 176.1)	-19.6 (-69.1; 30.0)	0.437
Sys blood pressure (mm/Hg)	249	134.8 (14.9)	-4.7 (-6.7; -2.6)	<0.001
Dia blood pressure (mm/Hg)	249	82.8 (10.2)	-4.0 (-5.5; -2.5)	<0.001
Triglycerides (mmol/L)	237	1.2 (0.9-1. 7)	-0.18 (-0.27; -0.09)	<0.001
Total Cholesterol (mmol/L)	234	5.4 (1.4)	-0.24 (-0.38; -0.11)	<0.001
HDL cholesterol (mmol/L)	235	1.3 (0.4)	0.00 (-0.04; 0.03)	0.780
**Aerobic fitness**				
VO_2_max (ml/kg/min)	257	29.0 (7.7)	2.8 (2.2; 3.4)	<0.001


*Data are means (SD), medians (interquartile range) or estimated changes (95% CI). P: p-value for overall unadjusted test of change.*

### Variability in Individual Responses

**FIGURE 1:**
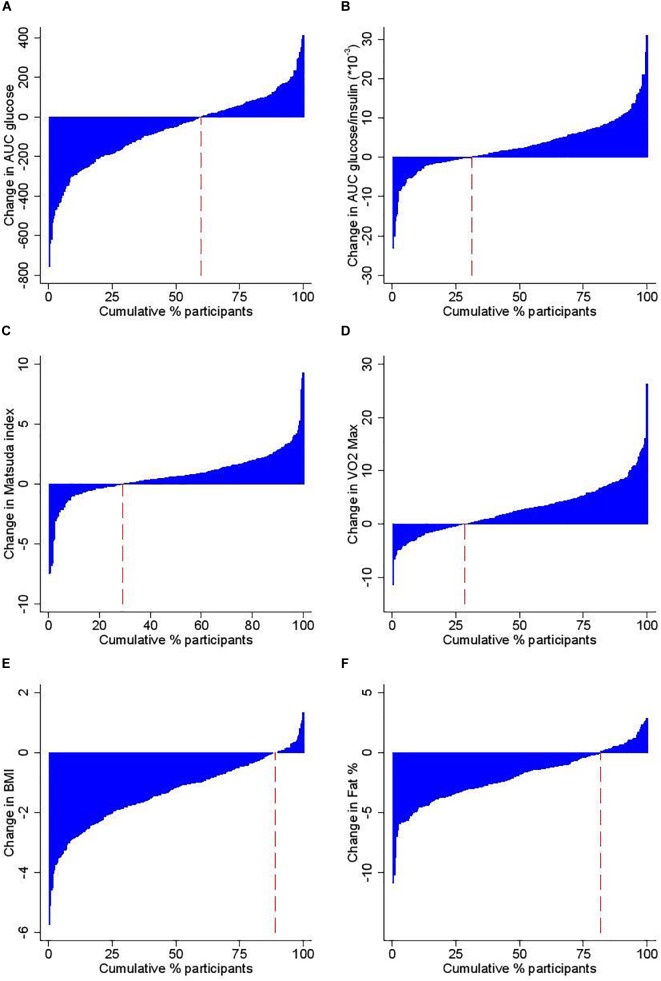
Study participants according to change following the 12 week intervention in **(A)** Area Under the Curve (AUC) for Glucose during the OGTT, **(B)** AUC for Glucose/Insulin during the OGTT, **(C)** insulin sensitivity as estimated by the Matsuda index, **(D)** VO_2_max, **(E)** BMI, and **(F)** % body fat. The red dashed line indicates the proportion of individuals with a lowered outcome level after the intervention.

While the overall responses to the intervention were positive, there was a broad range of individual responses in the measured parameters, as shown in the waterfall plots (Figure 1). BMI and body fat decreased in 80–90% of participants while 70% increased VO_2max_. Fasting and 2 h glucose as well as AUC_glucose_ decreased in 60–64% of participants while insulin sensitivity improved in ∼70% of participants.

### Glycemic Status at Baseline and Response to the Intervention

Participants were divided into those with (i) NGT (32.6%), (ii) i-IFG (40.8%), (iii) IGT (isolated IGT and combined IFG& IGT: 17.3%), and (iv) T2DM (9.3%). Participants were excluded if they had diabetes at the time of recruitment but a number were subsequently found to have screen-detected diabetes at the first OGTT. There was an overall improvement in the response, as determined by the difference between pre- and post-intervention scores, for each group (Table 2). After adjusting for age, gender and BMI, differences between baseline glycaemic status groups were identified in the fasting and 2 h blood glucose responses. With additional adjustment for baseline variability, there were no differences in the response between groups (Table 2).

**Table 2:** Estimated impact of the intervention (imputed data) by glycemic group.

	NGT (*n* = 84)	i-IFG (*n* = 105)	i-IGT and IFG/IGT (*n* = 44)	T2DM (*n* = 24)	*P*_1_	*P*_2_
**Body composition**						
Weight (kg)	-4.0 (-4.7; -3.3)	-3.6 (-4.4; -2.9)	-3.3 (-4.4; -2.3)	-5.0 (-6.4; -3.6)	0.471	0.469
BMI (kg/m^2^)	-1.4 (-1.6; -1.1)	-1.3 (-1.5; -1.0)	-1.1 (-1.5; -0.8)	-1.7 (-2.2; -1.3)	0.248	0.359
Waist circumference (cm)	-6.0 (-7.5; -4.5)	-5.0 (-6.7; -3.2)	-4.8 (-7.1; -2.5)	-5.8 (-9.0; -2.7)	0.692	0.877
Fat (%)	-1.9 (-2.3; -1.4)	-2.0 (-2.5; -1.5)	-1.8 (-2.5; -1.1)	-2.8 (-3.7; -1.9)	0.240	0.349
Subcutanous fat (cm)	-0.38 (-0.57; -0.18)	-0.38 (-0.58; -0.17)	-0.59 (-0.87; -0.30)	-0.43 (-0.8; -0.07)	0.648	0.572
Visceral fat (cm)	-1.24 (-1.71; -0.76)	-0.74 (-1.26; -0.22)	-0.91 (-1.66; -0.16)	-1.27 (-2.2; -0.35)	0.701	0.621
**Clinical measurements**						
Fasting plasma glucose (mmol/L)	-0.30 (-0.42; -0.18)	0.08 (-0.05; 0.22)	-0.14 (-0.32; 0.05)	-0.93 (-1.18; -0.67)	<0.001*	0.055
2 h plasma glucose (mmol/L)	-0.08 (-0.42; 0.25)	-0.04 (-0.42; 0.34)	-1.62 (-2.14; -1.10)	-1.50 (-2.24; -0.76)	< 0.001^∞^	0.306
Fasting Insulin (pmol/l)	-11.8 (-31.6; 8.0)	10.9 (-11.3; 33.2)	-21.5 (-51.8; 8.8)	-33.8 (-75.2; 7.6)	0.110	0.284
AUC glucose (mmol min/L)	-48.2 (-82.2; -14.1)	0.5 (-37.9; 38.9)	-149.9 (-202.9; -96.9)	-203.9 (-277.2; -130.6)	<0.001^¥^	0.985
AUC insulin (⋅10^3^ pmol min/L)	-21.0 (-27.8; -14.3)	-13.3 (-20.8; -5.7)	-23.5 (-33.9; -13.1)	-26.3 (-40.4; -12.2)	0.508	0.529
Matsuda index	0.88 (0.49; 1.26)	0.38 (-0.06; 0.81)	0.66 (0.07; 1.25)	1.37 (0.56; 2.17)	0.345	0.278
Insulinogenic Index	-22.2 (-105.9; 61.4)	-27.6 (-85.6; 30.4)	-2.4 (-81.1; 76.3)	-42.7 (-152.0; 66.6)	0.824	0.736
Systolic blood pressure (mmHg)	-4.1 (-7.3; -1.0)	-5.5 (-9.0; -1.9)	-5.4 (-10.3; -0.5)	-1.0 (-7.5; 5.5)	0.505	0.129
Diastolic blood pressure (mmHg)	-3.4 (-5.7; -1.1)	-5.1 (-7.8; -2.5)	-3.2 (-6.8; 0.4)	-4.4 (-9.3; 0.5)	0.832	0.921
Triglycerides (mmol/L)	-0.25 (-0.39; -0.11)	-0.10 (-0.26; 0.05)	-0.10 (-0.32; 0.13)	-0.30 (-0.59; -0.01)	0.566	0.158
Total cholesterol (mmol/L)	-0.27 (-0.47; -0.07)	-0.11 (-0.33; 0.11)	-0.44 (-0.75; -0.12)	-0.23 (-0.65; 0.20)	0.326	0.627
HDL cholesterol (mmol/L)	0.01 (-0.04; 0.06)	0.00 (-0.06; 0.05)	-0.05 (-0.13; 0.03)	0.01 (-0.10; 0.11)	0.626	0.427
**Aerobic fitness**						
VO_2_max (ml/kg/min)	3.2 (2.3; 4.2)	2.7 (1.6; 3.8)	2.4 (1.0; 3.9)	2.0 (0.0; 4.0)	0.535	0.238


*Data are estimated changes (95% CI).*

*P_*1*_, p-value for overall test of difference in change between subgroups of glycaemia, adjusted for age, sex, BMI; P_*2*_, additional adjusting for the baseline level of the covariate.*

*^∗^Fasting plasma glucose (mmol/L): i-IFG vs. NGT: p < 0.001; T2DM vs. other groups: p < 0.001.*

*^∞^2 h plasma glucose (mmol/L): IGT vs. NGT: p < 0.001; IGT vs. i-IFG: p < 0.001; T2DM vs. NGT: p < 0.001; T2DM vs. i-IFG: p < 0.001.*

*^¥^AUC glucose (mmol min/L): IGT vs. NGT: p < 0.001; IGT vs. i-IFG: p < 0.001; T2DM vs. NGT: p < 0.001; T2DM vs. i-IFG: p < 0.001.*

### Cluster Analysis Based on Change in Area Under the Curve for Glucose

A hierarchical cluster analysis based on the change in the AUC_glucose_ identified four sub-groups, which could be categorized as either responders or non-responders (Table 3). Cluster 1 (*n* = 17) and Cluster 2 (*n* = 57) showed a High (HI-RES) or Moderate (MOD-RES) improvement in AUC_glucose_ while Cluster 3 (*n* = 126) and Cluster 4 (*n* = 57) did not show any change (NO-RES) or had deteriorated (DET-RES) AUC_glucose_. Almost 50% of the high responders (Cluster 1) had T2DM while those with i-IFG formed the largest proportion in the moderate responder (Cluster 2) and no response (Cluster 3) groups. Of those that deteriorated (Cluster 4) just under 50% had NGT (Table 3).

**Table 3:** Response to the intervention based on clusters of change in AUC_glucose_ (imputed data).

	Responders		Non-responders			
				
Baseline	C1 HI-RES	C2 MOD-RES	C3 NO-RES	C4 DET-RES	*P*_1_	*P*_2_
N	17	57	126	57		
Mean (SD) of change in AUC_glucose_	-485 (101)	-236 (52)	-34 (63)	161 (82)		
Range of change in AUC_glucose_	-756; -368	-367; -157	-156; 70	71; 401		
**Baseline**						
Age (year)	53.4 (11.0)	53.6 (11.5)	52.9 (11.2)	57.9 (8.7)		
Male sex (%)	65	53	51	40		
NGT (%)	6	18	37	47		
i-IFG (%)	18	42	44	39		
IGT (%)	29	26	15	7		
T2DM (%)	47	14	4	7		
**Change**						
**Body composition**						
Weight (kg)	-7.5 (4.7)	-4.4 (3.4)^a^	-3.6 (3.3)^a^	-2.7 (2.8)^a,b^	<0.001	<0.001
BMI (kg/m^2^)	-2.6 (1.5)	-1.5 (1.1)^a^	-1.2 (1.1)^a^	-1.0 (1.0)^a,b^	<0.001	<0.001
Waist circumference (cm)	-9.7 (6.7)	-5.2 (6.9)	-4.6 (7.6)	-4.8 (6.6)	0.092	0.058
Fat (%)	-3.8 (2.3)	-2.5 (2.4)^a^	-1.7 (2.2)^a,b^	-1.6 (1.7)^a,b^	<0.001	<0.001
Subcutanous fat (cm)	-0.68 (1.0)	-0.59 (0.72)	-0.37 (0.95)	-0.40 (0.80)	0.372	0.172
Visceral fat (cm)	-2.17 (2.41)	-1.27 (2.06)	-0.84 (2.22)	-0.91 (1.96)	0.137	0.566
**Clinical measurements**						
Fasting plasma glucose (mmol/L)	-0.74 (1.14)	-0.48 (0.81)	-0.11 (0.47)	0.02 (0.59)^a,b,c^	<0.001	0.010
2 h plasma glucose (mmol/L)	-3.7 (1.9)	-1.6 (1.2)^a^	-0.3 (1.1)^a,b^	1.1 (1.8)^a,b,c^	<0.001	<0.001
AUC glucose (mmol min/L)	-484 (113)	-239 (56)^a^	-35 (65)^a,b^	155 (72)^a,b,c^	<0.001	<0.001
AUC insulin (⋅10^3^ pmol min/L)	-60.2 (54.1)	-31.4 (38.0)^a^	-16.1 (26.7)^a,b^	-2.9 (25.7)^a,b,c^	<0.001	<0.001
Matsuda index	2.47 (2.61)	1.45 (1.85)	0.53 (1.62)^a,b^	-0.11 (2.14)^a,b^	<0.001	<0.001
Insulinogenic index	17.7 (44.2)	31.8 (121.8)	-31.9 (149.1)	-55.1 (204.7)^b^	0.038	0.041
Systolic blood pressure (mmHg)	-1.2 (11.3)	-5.1 (17.2)	-5.4 (15.6)	-3.6 (16.1)	0.761	0.139
Diastolic blood pressure (mmHg)	-3.0 (10.5)	-4.4 (12.1)	-3.7 (12.3)	-4.5 (11.0)	0.957	0.735
Total cholesterol (mmol/L)	-0.71 (1.08)	-0.35 (0.97)	-0.16 (1.05)	-0.17 (0.82)	0.186	0.113
Triglycerides (mmol/L)	-0.56 (1.02)	-0.26 (0.66)	-0.15 (0.70)	-0.08 (0.52)	0.090	0.930
HDL cholesterol (mmol/L)	-0.01 (0.21)	0.01 (0.26)	0.02 (0.26)	-0.06 (0.22)	0.382	0.465
**Fitness and physical activity**						
VO_2_max (ml/kg/min)	3.8 (8.0)	2.7 (3.7)	3.1 (4.6)	1.9 (4.5)	0.359	0.304
Exercise time (mins)	3604 (2035; 5827)	2585 (1485; 3560)	2318 (1271; 3615)	2120 (1328; 3550)	0.123	–


*Data are estimated changes (95% CI). P_*1*_, p-value for overall unadjusted test of difference between clusters. P_*2*_, overall test adjusted for the baseline level of the covariate.*

*Pairwise tests of difference between clusters adjusted for the baseline level of the covariate: ^*a*^P < 0.05 vs. C1.*

*^*b*^P < 0.05 vs. C2.*

*^*c*^P < 0.05 vs. C3.*

The physiological and clinical characteristics of participants in the four clusters are presented in terms of their response to the intervention (Table 3) and their baseline data prior to the intervention (Table 4). There were few differences in baseline characteristics between the clusters. DET-RES (Cluster 4) had lower body weight at baseline compared with HI-RES (Cluster 1) and NO-RES (Cluster 3) but not MOD-RES (Cluster 2). HI-RES (Cluster 1) had higher visceral fat and triglycerides than the other clusters while HDL Cholesterol was higher in DET-RES (Cluster 4) than the groups that responded (Cluster 1 and 2). There were no differences in total body fat, BMI, waist circumference, VO_2max_ or subcutaneous body fat. The non-responder groups (Cluster 3 and 4) had better baseline glycemic characteristics than those that responded (Clusters 1 and 2), as expected (Table 4).

**Table 4:** Baseline characteristics based on the clusters of change in AUC_glucose_ (imputed data).

	Responders		Non-responders		
				
	C1 HI-RES	C2 MOD-RES	C3 NO-RES	C4 DET-RES	*P*
N	17	57	126	57	
Mean (SD) of change in AUC_glucose_	-485 (101)	-236 (52)	-34 (63)	161 (82)	
Range of change in AUC_glucose_	-756; -368	-367; -157	-156; 70	71; 401	
Age (year)	53.4 (11.0)	53.6 (11.5)	52.9 (11.2)	57.9 (8.7)	
Male sex (%)	65	53	51	40	
NGT (%)	6	18	37	47	
i-IFG (%)	18	42	44	39	
IGT (%)	29	26	15	7	
T2DM (%)	47	14	4	7	
**Body composition**					
Weight (kg)	96.0 (13.7)	89.5 (16.5)	91.3 (18.9)	84.2 (17.2) ^a,c^	0.038
BMI (kg/m^2^)	33.4 (4.1)	30.9 (4.9)	31.2 (5.6)	30.4 (6.0)	0.262
Waist circumference (cm)	111.2 (11.3)	103.2 (10.7)	105.1 (12.3)	101.9 (14.0)	0.070
Fat (%)	39.7 (6.9)	37.9 (8.8)	37.2 (8.4)	38.4 (9.4)	0.656
Subcutanous fat (cm)	3.0 (1.2)	3.0 (1.3)	2.9 (1.2)	2.7 (1.0)	0.679
Visceral fat (cm)	9.4 (2.1)	7.5 (2.3)^a^	7.0 (2.1)^a^	7.0 (2.2)^a^	0.002
**Clinical measurements**					
Fasting plasma glucose (mmol/L)	6.6 (1.7)	6.2 (1.0)	5.7 (0.6)^a,b^	5.8 (0.7)^a,b^	<0.001
2 h plasma glucose (mmol/L)	10.3 (3.7)	7.7 (2.3)^a^	6.3 (1.7)^a,b^	6.2 (1.5)^a,b^	<0.001
AUC glucose (mmol min/L)	1668 (469)	1332 (295)^a^	1127 (214)^a,b^	1118 (220)^a,b^	<0.001
AUC insulin (⋅10^3^ pmol min/L)	110.0 (89.1; 128.9)	76.7 (58.8; 122.8)	71.5 (45.9; 103.4)^a^	61.0 (42.5; 91.2)^a,b^	0.006
Matsuda index	1.7 (1.3; 2.3)	2.3 (1.7; 3.9)^a^	3.0 (2.1; 4.7)^a,b^	3.6 (2.8; 5.3)^a,b^	<0.001
Insulinogenic index	112.7 (68.9; 130.4)	113.5 (66.7; 168.1)	124.8 (83.8; 203.2)	103.5 (69.3; 185.9)	0.162
Systolic blood pressure (mmHg)	141.6 (12.9)	136.5 (16.6)	133.7 (13.4)	133.5 (16.6)	0.174
Diastolic blood pressure (mmHg)	84.6 (8.5)	83.3 (10.6)	82.3 (10.4)	82.6 (10.0)	0.832
Total cholesterol (mmol/L)	5.5 (1.0)	5.3 (1.3)	5.4 (1.6)	5.3 (1.0)	0.958
Triglycerides (mmol/L)	1.8 (1.3; 2.8)	1.4 (0.9; 1.9)^a^	1.2 (0.9; 1.6)^a^	1.1 (0.8; 1.5)^a,b^	0.004
HDL cholesterol (mmol/L)	1.1 (0.2)	1.2 (0.3)	1.3 (0.4)	1.4 (0.3)^a,b^	0.007
**Fitness and physical activity**					
VO_2_max (ml/kg/min)	28.8 (6.2)	29.8 (7.3)	29.2 (8.2)	28.0 (7.5)	0.635
Exercise time (mins)	3604 (2035; 5827)	2585 (1485; 3560)	2318 (1271; 3615)	2120 (1328; 3550)	0.123


*Data are means (SD) or medians (interquartile range). P, p-value for overall unadjusted test of difference between clusters. Skewed distribution data were log-transformed prior to the test.*

*^*a*^P < 0.05 vs. C1.*

*^*b*^P < 0.05 vs. C2.*

*^*c*^P < 0.05 vs. C3.*

The change in each variable following the intervention was also assessed to determine if the cluster analysis could identify physiological or clinical characteristics to differentiate the groups (Table 3). Cluster 1 (HI-RES) lost more weight and body fat than the other groups but there were no differences between cluster 2 (MOD-RES) and cluster 3 (NO-RES). All groups improved to a similar degree in waist circumference, abdominal fat, blood pressure, lipids and VO_2max_. There was no significant difference in the number of minutes of exercise completed during the intervention.

### Principal Component Analysis of Individual Variability

**FIGURE 2:**
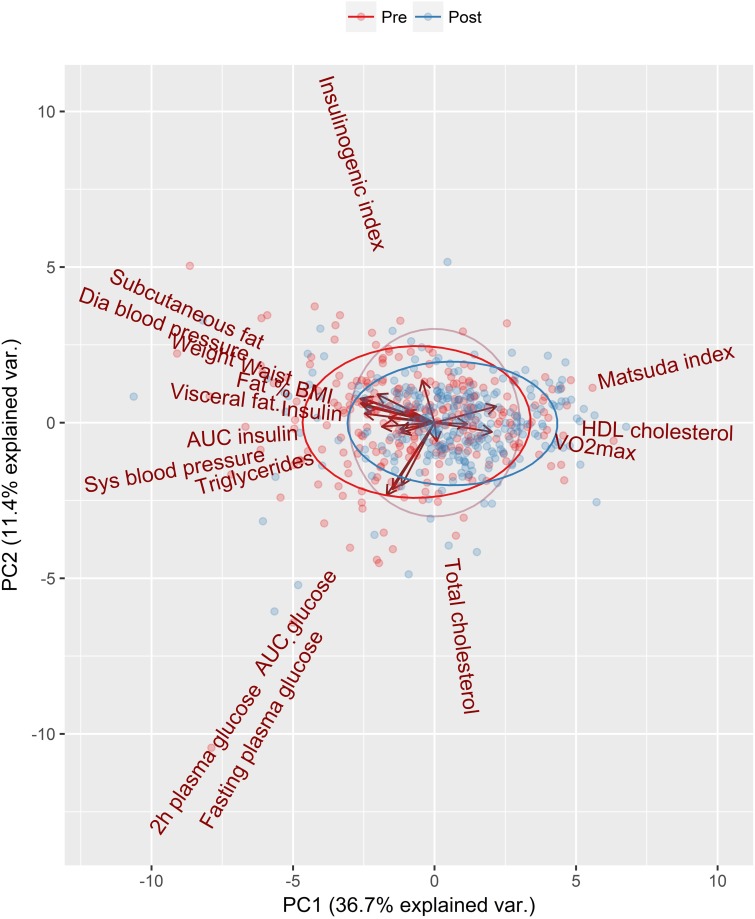
The first two principal components of the data, showing a multivariate change over the 12 weeks intervention. The first and second principal components (PC1 and PC2) are shown on the x-axis and y-axis, respectively. The loadings of the variables are shown with arrows, and the scores of the participants are shown as dots in the background with red (pre) and blue (post-intervention). Arrows pointing to the right (on the x-axis) and upwards (on the y-axis) illustrate a positive change score, e.g., Matsuda index and VO_2_max (l/min). Arrows pointing to the left (on the x-axis) and downwards indicate a negative change score.

PCA was applied to explore individual variation in response using all measured clinical and physiological parameters (Figure 2). This multivariate visualization is complementary to the univariate waterfall plots. There was a rightward shift in standard deviation ellipses that represent the overall variance, indicating a positive response to the intervention. Individual changes in all measured parameters (*n* = 19) were subjected to PCA resulting in a two component solution that only accounted for 48% of total variation. The loadings are presented in Table 5. The highest loadings in the first principal component (PC1), which explained 36.7% of variation, were BMI (-0.33), waist circumference (-0.33) and visceral fat (-0.31). The highest loadings in the second principal component (PC2), comprising 11.4% of total variation, were AUC_glucose_ (-0.52) and 2 h blood glucose (-0.48), fasting plasma glucose (0.45), and insulinogenic index (0.31).

**Table 5:** Loading of the variables in the two principal components, sorted by the magnitude of the loadings in PC 1.

Variable	PC1	PC2
Waist circumference	-0.330	0.150
BMI	-0.328	0.142
Weight	-0.318	0.175
% fat	-0.316	0.122
Visceral fat	-0.310	0.0675
Matsuda index	0.274	0.118
VO_2max_ ml/kg/min	0.255	-0.0664
Subcutaneous fat	-0.254	0.206
AUC insulin	-0.234	-0.0281
AUC glucose	-0.213	-0.524
Insulin	-0.202	0.0343
Blood glucose_120 min	-0.187	-0.481
BP Systolic	-0.152	-0.0484
Triglycerides	-0.148	-0.0717
Fasting plasma glucose	-0.145	-0.452
Cholesterol (HDL)	0.144	-0.0089
BP Diastolic	-0.128	0.0833
Insulinogenic index	-0.0540	0.306
Cholesterol (total)	-0.0097	-0.135


## Discussion

The main findings from this study confirm the overall positive impact of a lifestyle intervention on a group at high risk of developing type 2 diabetes (Tuomilehto et al., 2001; Knowler et al., 2002; Lindström et al., 2006; Ramachandran et al., 2006; Li et al., 2008) but highlight the challenges identifying sub-groups of individuals that are likely to respond or not respond. Our data demonstrate that when participants were categorized by glycemic status or changes in glucose tolerance following the intervention, it was not possible to identify a set of phenotypic characteristics that could differentiate sub-groups.

Following the 12 weeks intervention, most subjects had decreased BMI, waist circumference and body fat (80–89%) and increased aerobic fitness (72%). However, in agreement with others (Solomon et al., 2013; Stefan et al., 2015), we found a lesser proportion of subjects with improved fasting plasma glucose (64%), 2 h glucose (60%), and AUC glucose (62%). Sparks (2017) argues that a sizeable proportion of individuals do not respond to exercise training and the outcome depends on the variable selected. It is also possible that changes in total daily activity or sedentary time over the course of the intervention might play a role. However, there are many factors that can influence the inter-individual variability in response to a lifestyle intervention (Solomon, 2018) and it is still a matter of discussion whether variables should be isolated and their contribution analyzed or if it would be more effective to identify molecular or metabolic biomarkers to collectively account for the overall variance.

Previous research has suggested the differences in response to a lifestyle intervention could be due to the inclusion of different prediabetic glycemic categories (Schafer et al., 2007; Malin et al., 2012, 2013; Solomon et al., 2015) since the pathophysiology of progression to type 2 diabetes may differ based on the glycemic status (Faerch et al., 2016). However, despite differences in the baseline glycemic status of our participants, the average improvement was similar between groups and comparable with diabetes prevention studies in the literature (Tuomilehto et al., 2001; Lindström et al., 2006). These data are supported by Malin et al. (2012, 2013) who found no differences in the responses between glycemic groups to an exercise intervention (Malin et al., 2012) and that the change in fasting plasma glucose was the only variable to differ between groups (Malin et al., 2013). Similar results have been reported for a 9 month intervention (Schafer et al., 2007) where NGT and IGT subjects decreased body weight, visceral and liver fat with improved insulin sensitivity. Collectively, these findings demonstrate a similar clinical and metabolic responses to a lifestyle intervention in different glycemic groups.

The cluster analysis identified four distinct sub-groups including high (HI-RES) and moderate (MOD-RES) responders, a group that were unchanged (NO-RES) and one that had deteriorated (DET-RES) glucose tolerance. Other studies found that 15–20% of individuals with type 2 diabetes do not improve glucose tolerance (Sparks et al., 2013) and that ∼30% with IGT/T2DM do not change blood glucose following exercise training (Solomon et al., 2013; Stefan et al., 2015). Our results are comparable despite the larger number of NGT and i-IFG subjects in the present study. The baseline characteristics were similar between the four clusters, despite differences in AUC_glucose_ following the intervention. Those that deteriorated (Cluster 4) had a lower body weight, triglycerides and HDL cholesterol but were not sufficient to identify a set of characteristics that would predict a change in glucose tolerance following a lifestyle intervention.

The main differences in response to the intervention were noted between the HI-RES group (Cluster 1) who achieved the greatest amount of weight loss but only accounted for 6% of subjects (Table 3). Approximately 75% of the participants fell into the MOD-RES (Cluster 2) and NO-RES (Cluster 3) groups. Apart from body fat there were no differences in the clinical or physiological variables between these two groups. The DET-RES group had a smaller weight and body fat reduction than the HI-RES and MOD-RES groups. However, these “non-responders,” in terms of glucose tolerance, still had an improvement in all clinical and physiological variables, highlighting the difficulties differentiating individuals most likely to improve their glycemia. It was notable that there was a greater proportion of men in the HI-RES cluster (65%) and women in the DET-RES cluster (60%), with similar sex distribution in MOD-RES and NO-RES. Further research will be required to determine if men and women are more likely to respond to a lifestyle intervention but we cannot rule out a potential confounding effect on the findings.

Other possible explanations for variability of response phenotypes have included baseline insulin secretory capacity (Solomon et al., 2013), insulin resistance or the presence of non-alcoholic fatty liver disease (Stefan et al., 2015, 2016). Using estimates derived from the OGTT we did not observe differences in the response of insulin sensitivity or insulin secretion between the glycemic groups, however, a hyperglycemic clamp is more sensitive than an OGTT in this regard. The 4 clusters were based on change in glucose tolerance so it is not surprising the two “responder” groups were more insulin sensitive. There was no pattern in the insulinogenic index at baseline, or in response to the intervention, to suggest a primary role for compromised insulin secretion.

Principal Component Analysis was used to summarize the multivariate data. The standard deviation ellipses support the overall positive group response with the observed shift to the right (Figure 2). All parameters were included in this analysis, yet only 48% of the variance could be explained. The changes in body weight or composition are often reported to demonstrate the effectiveness of a lifestyle intervention, or explain the response, but PC1, made up mostly of body composition variables, only accounted for 37% of the overall variance. PC2 contained a mix of variables linked to glucose tolerance and VO_2_max that only accounted for ∼11% of the variance.

These findings are consistent with several published studies (Thamer et al., 2007; Totsikas et al., 2011; Solomon et al., 2013; Mann et al., 2014). Solomon et al. (2013) reported correlations between changes in insulin secretion and changes in glycemia that explain ∼6–16% of the variance. Thamer et al. (2007) found associations with BMI, visceral adipose tissue and leg fat that explain ∼8–16% of the variance in insulin sensitivity, while Totsikas et al. (2011) found an association between a high anaerobic threshold at baseline and the prediction of improvements in insulin sensitivity that explained ∼4% of the variation. Thus, while there are significant associations between changes in phenotypic characteristics and glycemic outcomes, only small amounts of the overall variance can be explained; and even when combined in multivariate analysis still less than half of the variance is accounted for, highlighting the need for better predictors of improvements in glycemic status.

## Conclusion

In conclusion, we report a broad range of individual responses in individuals at high risk for type 2 diabetes following a 12 weeks lifestyle intervention. We found that the standard clinical and physiological variables were not sufficient to predict the responsiveness to an intervention in the majority of individuals. In agreement with Solomon (2018) we believe there is a need for additional biomarkers to complement standard clinical measures that help predict blood glucose responses to a lifestyle intervention.

## Data Availability

All datasets generated for this study are included in the manuscript and/or the Supplementary Files.

## Author Contributions

JJN and DO’G conceived and designed the study. GO’D and AK were involved in trial project management, recruitment, and acquisition of all data. SC, ED, HD, PF, HK, NM, EM, JN, MW were involved in data acquisition. GO’D, DO’G, and JJN wrote the manuscript. AK and KF researched data and contributed to the discussion. GA, DV, and TS performed the statistical analysis. All authors reviewed and edited the manuscript. DO’G was the guarantor of this work and, as such, had full access to all the data in the study and takes responsibility for the integrity of the data and the accuracy of the data analysis.

## Conflict of Interest Statement

The authors declare that the research was conducted in the absence of any commercial or financial relationships that could be construed as a potential conflict of interest.

## Footnotes

1 www.R-project.org
