# Supplementary material for: Phenotypic Responses to a Lifestyle Intervention Do Not Account for Inter-Individual Variability in Glucose Tolerance for Individuals at High Risk of Type 2 Diabetes
Source: Front Physiol. 2019 Mar 26;10:317. doi: 10.3389/fphys.2019.00317 (PMC6443958; doi:10.3389/fphys.2019.00317)
Supplement: Supplementary file 1 [file Table_1.DOCX]

**Supplementary Table 1:** Baseline characteristics of the control group (original data) and estimated impact of intervention (imputed data)

|  | **Original data** | | **Imputed data** | |
| --- | --- | --- | --- | --- |
|  | **n** | **Level** | **Change (95% CI)** | **P** |
| Age (year) | 78 | 55.0 (12.2) |  |  |
| Male sex (%) | 80 | 50.0 |  |  |
|  |  |  |  |  |
| **Body Composition** |  |  |  |  |
| Weight (kg) | 79 | 89.6 (77.7) | -0.4 (-1.2;0.3) | 0.232 |
| BMI (kg/m^2^) | 79 | 30.5 (28.0) | -0.2 (-0.4;0.1) | 0.226 |
| Waist (cm) | 76 | 104.1 (97.1) | -1.9 (-3.6;-0.2) | 0.027 |
| Fat % | 80 | 37.2 (32.1) | -0.1 (-0.6;0.4) | 0.723 |
| Subcutaneous fat (cm) | 75 | 3.0 (2.2;3.9) | -0.21 (-0.42;0.00) | 0.054 |
| Visceral fat (cm) | 74 | 7.4 (5.7;8.7) | -0.06 (-0.56;0.44) | 0.804 |
|  |  |  |  |  |
| **Clinical Measurements** |  |  |  |  |
| Fasting plasma glucose (mmol/l) | 78 | 5.6 (0.6) | 0.03 (-0.12;0.18) | 0.690 |
| 2h plasma glucose (mmol/l) | 77 | 6.5 (1.9) | -0.05 (-0.46;0.37) | 0.821 |
| Insulin (pmol/l) | 76 | 75.5 (57.4;109,6) | 2.45 (-18.45;23.34) | 0.818 |
| AUC glucose (mmol min/L) | 70 | 1078 (956;1286) | 8.9 (-32.3;50.1) | 0.671 |
| AUC insulin (∙10^3^ pmol min/L) | 72 | 74.3 (49.1;102.7) | -1.9 (-9.4;5.7) | 0.630 |
| Matsuda index | 71 | 3.7 (2.7) | -0.14 (-0.57;0.28) | 0.512 |
| Insulinogenic index | 80 | 147.3 (74.3;225.5) | -53.3 (-241.4;134.7) | 0.569 |
| Sys Blood Pressure (mm/Hg) | 77 | 134.3 (14.3) | -3.7 (-7.5;0.1) | 0.055 |
| Dia Blood pressure (mm/Hg) | 76 | 84.1 (9.7) | -3.0 (-5.7;-0.3) | 0.032 |
| Triglycerides (mmol/L) | 75 | 1.3 (0.9;1.8) | -0.04 (-0.21;0.13) | 0.635 |
| Total Cholesterol (mmol/L) | 74 | 5.2 (1.4) | 0.05 (-0.19;0.29) | 0.693 |
| HDL cholesterol (mmol/L) | 74 | 1.3 (0.4) | 0.00 (-0.06;0.06) | 0.950 |
|  |  |  |  |  |
| **Aerobic Fitness** |  |  |  |  |
| VO_2_max (ml/kg/min) | 79 | 28.2 (7.4) | 0.7(-0.4;1.7) | 0.220 |

Data are means (SD), medians (interquartile range) or estimated changes (95% CI). P: p-value for overall unadjusted test of change
